# Maternal and fetal outcomes in gestational diabetes mellitus: a narrative review of dietary interventions

**DOI:** 10.3389/fgwh.2025.1510260

**Published:** 2025-02-26

**Authors:** Anuja Phalle, Devaki Gokhale

**Affiliations:** Department of Nutrition & Dietetics, Symbiosis School of Culinary Arts & Nutritional Sciences, Symbiosis International (Deemed) University, Pune, Maharashtra, India

**Keywords:** gestational diabetes mellitus, dietary regimens, ramadan fasting, DASH diet, calorie restriction, Mediterranean diet, plant-based diet, maternal outcomes

## Abstract

**Introduction:**

Gestational diabetes mellitus (GDM) is associated with a myriad of maternal and fetal complications that severely compromise the mother and child's future health. Dietary interventions are effective in reducing the risk of GDM. However, when diagnosed with GDM in 2nd and 3rd the effectiveness of these interventions on maternal and fetal health remains unexplored. Therefore, this review critically examines existing literature for short- and long-term maternal and fetal outcomes of dietary interventions followed after GDM diagnosis.

**Methodology:**

An extensive literature search through Scopus, PubMed, and Web of Science was conducted to include original, full-text articles published in English between 2013 and April 2024. All randomized controlled trials, case-control, prospective cohort studies, and longitudinal follow-up trials that recruited GDM mothers following dietary interventions upon diagnosis were included. However, pre-pregnancy interventional, retrospective, and prospective studies reporting maternal and fetal outcomes in healthy pregnant women were excluded. This review followed the Narrative Review Checklist by the Academy of Nutrition & Dietetics, Elsevier.

**Results:**

We reviewed the effects of eight popular dietary interventions on various short and long-term materno-fetal outcomes in women recently diagnosed with GDM. Dietary interventions such as Mediterranean, Dietary Approaches to Stop Hypertension (DASH), and low-GI positively affected both short and long-term maternal and fetal outcomes. In contrast, fasting during Ramadan negatively affected maternal and fetal outcomes. Studies with low-carb, high-protein, and calorie restriction reported mixed findings for materno-fetal outcomes. Although certain dietary interventions have shown beneficial effects in the past literature, their findings were limited by small sample size, short intervention duration, and inconsistencies in the outcomes and population studied, compromising the quality of evidence. Further, we observed a scarcity of studies exploring the effect of dietary interventions followed during 2nd and 3rd trimesters after being diagnosed with GDM on long-term materno-fetal outcomes.

**Conclusion:**

Dietary interventions followed during 2nd and 3rd trimesters after the diagnosis of GDM may be crucial for preventing short and long-term materno-fetal complications; however, there is a lack of strong evidence to support this notion. Future studies are recommended to monitor the long-term materno-fetal outcomes of GDM.

## Introduction

In recent years, Gestational diabetes mellitus (GDM) has become a common complication of pregnancy with a pooled prevalence of 14% across the globe based on the International Association of Diabetes and Pregnancy Study Group (IADPSG) criteria (1). Although IADPSG criteria are commonly used, they are consistently reported to predict a higher prevalence of GDM than any other criteria. However, regardless of the criteria, the pooled prevalence of GDM has been still higher (14.7%) globally (2). Region-wise, Middle-East North Africa (27.6%) and South-East Asia (20.8%) have the highest prevalence of GDM whereas, North America and the Caribbean (7.1%) and European regions (7.8%) have the lowest. Further, the burden of GDM is higher in high (14.2%) and low-income countries (12.7%) as compared to middle-income (9.2%) countries (1). GDM is the onset of diabetes characterized by insulin resistance and high blood glucose levels diagnosed between the 24th and 28th weeks of gestation (GW) during pregnancy (3, 4). GDM unlike Type I and II diabetes, is evident only in the later (2nd and 3rd) trimesters leaving a limited scope for early prevention. Furthermore, GDM has been associated with a myriad of negative short-term and long-term materno-fetal complications. GDM significantly increases the risk of non-communicable diseases in the future for both mother and fetus compromising their health status and quality of life (5–7).

Dietary intervention is a top-tiered lifestyle-based intervention for controlling blood glucose levels among women diagnosed with GDM. Past literature underscores the significance of pre-pregnancy adherence to Dietary Approaches to Stop Hypertension (DASH), the Mediterranean (MED) diet, and Plant-based/vegetarian diets in reducing the risk of developing GDM (8, 9). Adherence to these dietary interventions upon diagnosis of GDM may also help alleviate negative health consequences for mother and child. In the face of the ever-growing prevalence and adverse consequences of GDM, it becomes crucial to study high-quality evidence. This will facilitate a better understanding of dietary management during GDM to provide quality care to the mother and the growing fetus. However, the implications of adhering to these dietary interventions upon the diagnosis of GDM during 2nd and 3rd trimester on maternal and fetal outcomes have not been evaluated.

## Methods

This narrative review collates the existing evidence to understand the effects of following the popular dietary interventions on materno-fetal outcomes in women with GDM after diagnosis during 2nd and 3rd trimester.

**Figure 1:**
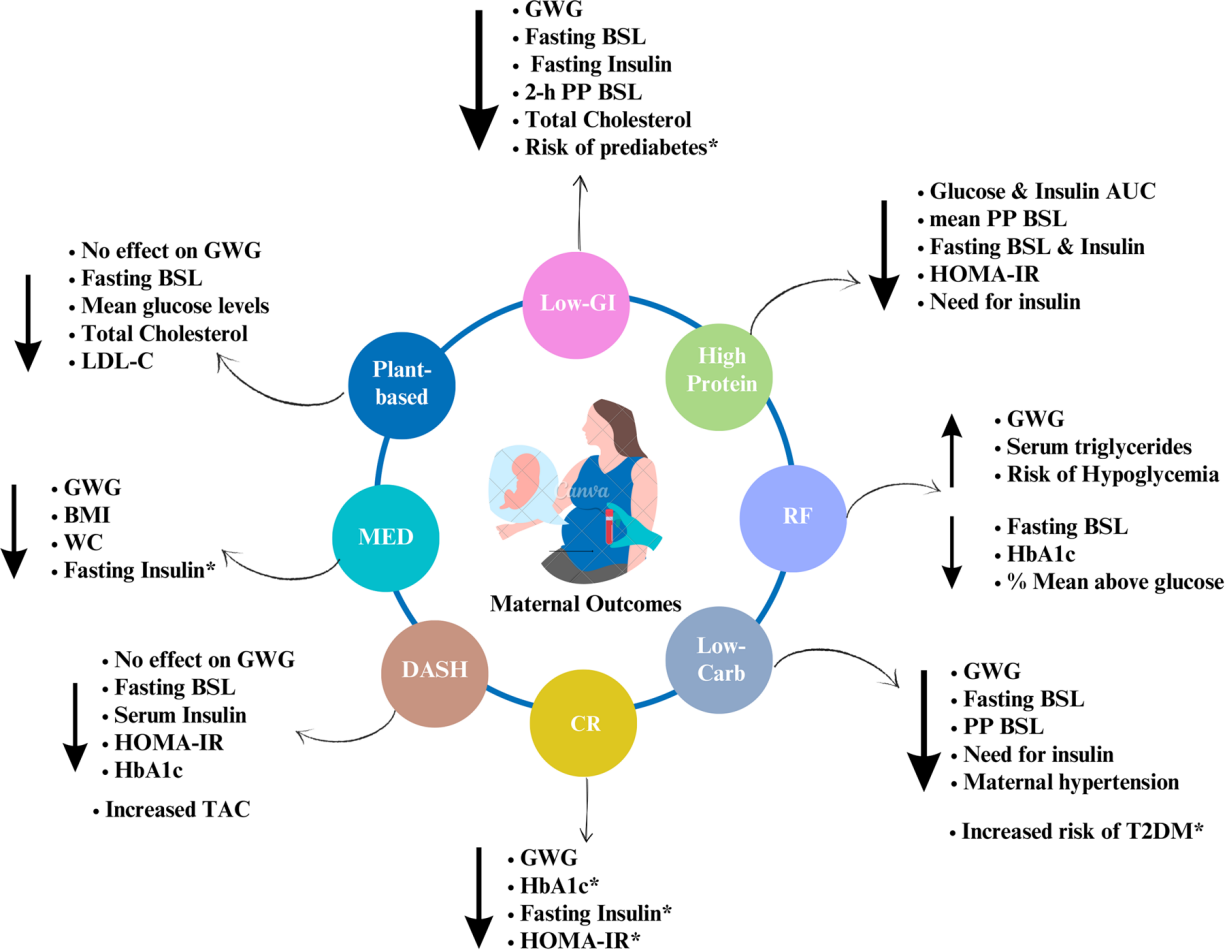
Summary of the effects of dietary interventions on maternal outcomes. Note: Author's creation using Canva software, *indicates the long-term effects; GWG, gestational weight gain; WC, waist circumference; BSL, blood sugar levels; PP, postprandial; AUC, area under curve; TAC, total antioxidant capacity; HOMA-IR, homeostasis model assessment of insulin resistance.

**Figure 2:**
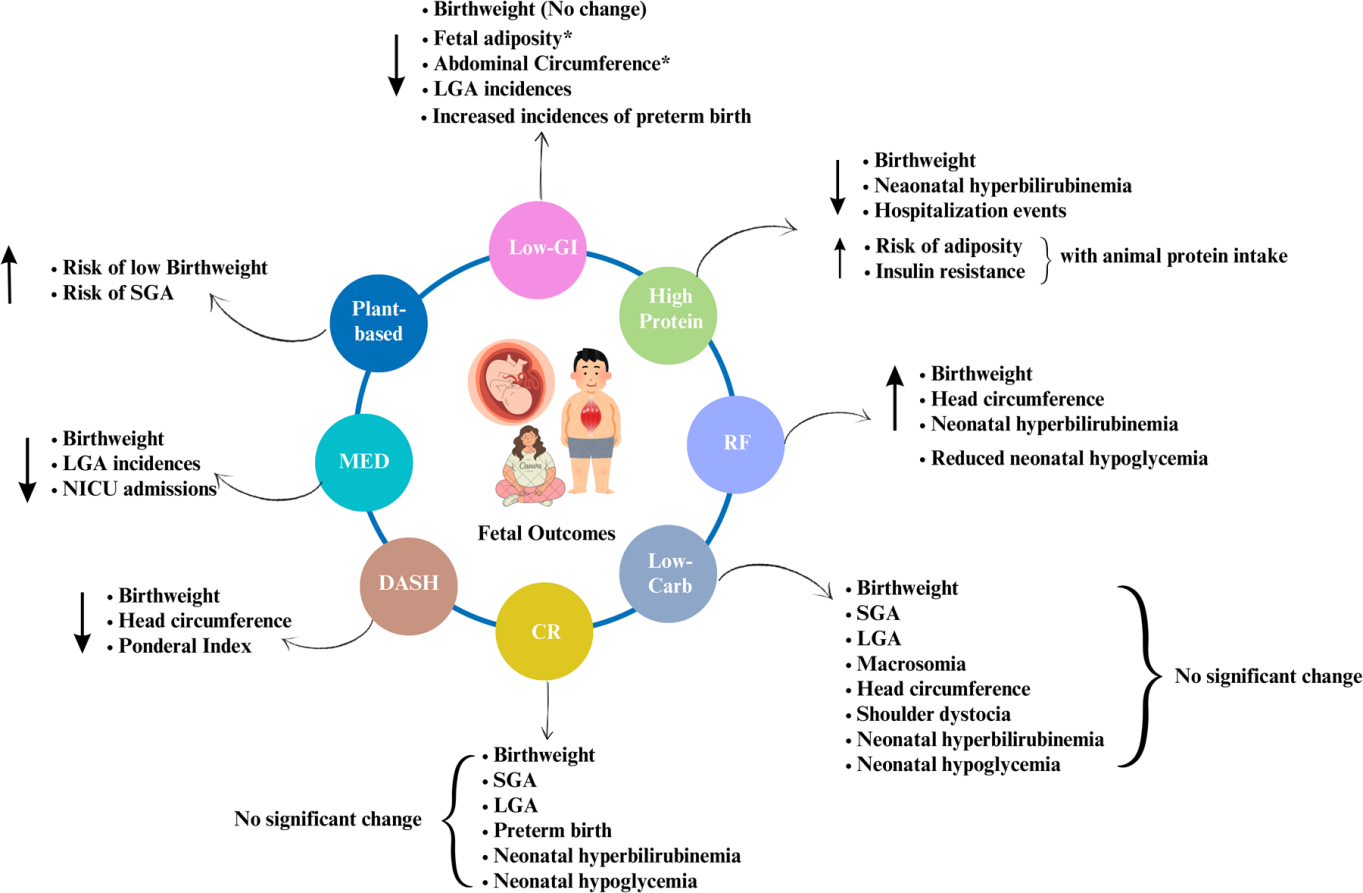
Summary of the effects of dietary interventions on fetal outcomes. Note: Author's creation using Canva software, *indicates the long-term effects; LGA, large for gestational age; SGA, small for gestational age.

The authors conducted an extensive search through Scopus and Web of Science databases using the keywords: “Mediterranean diet OR DASH diet OR plant-based diets OR Low Glycemic Index (GI) OR high fiber diet OR Ramadan Fasting OR Calorie Restriction OR Low-carbohydrate OR High protein Diet AND gestational diabetes mellitus.” All original, full-text articles published in English between 2013 and April 2024 were considered for this review. We reviewed the case-control, randomized controlled trials, prospective cohort, and longitudinal follow-up trials that investigated the effects of dietary interventions on short or long-term materno-fetal outcomes in women with GDM upon diagnosis during 2nd and 3rd trimesters. However, studies exploring the effects of dietary interventions followed during the pre-conception period on the risk of GDM and those recruiting healthy pregnant women were excluded. Therefore, this review exclusively focuses on mothers diagnosed with GDM, their adherence to various dietary interventions, and the short-term and future repercussions of these interventions on materno-fetal outcomes (Refer to Figure 1 and Figure 2 for summary of the findings). Both authors independently screened the articles for eligibility (Refer to Supplementary File 1 for details), and discrepancies were resolved by mutual discussion. Further, the reference lists of eligible studies were additionally screened to cover the literature extensively. The final review included a total of 61 articles, and the review followed the Narrative Review Checklist (10).

## Mediterranean diet (MED) and GDM

Mediterranean diet (MED) is a popular, plant-based dietary pattern consisting of large amounts of fruits, vegetables, cereals, nuts, and olive oil, moderate amounts of dairy products, fish, and poultry, and low amounts of processed meats and saturated fats prominently observed in Greece and southern Italy. has been shown to have positive maternal and fetal outcomes owing to supply of all essential nutrients crucial during pregnancy (11). Previously, pre-pregnancy adherence to MED has been widely investigated for its beneficial effects in reducing the risk of GDM, however, whether it has similar effects after diagnosis on materno-fetal outcomes has been less explored with only two studies meeting our inclusion criteria.

### Maternal outcomes

A secondary analysis of St. Carlos unicentric randomized controlled trial was done to evaluate the effectiveness of MED-based nutrition education among GDM vs. non-GDM women at 34–36th gestational week and 12–14 weeks post-partum. MED significantly prevented the excessive weight gain compared to the average weight gain in the GDM group at 36th–38th GW [RR 0.91 (0.86–0.96), *p* < 0.001]. Adherence to MED significantly lowered the gestational weight gain (GWG) post-delivery in the GDM (10.0 kg ± 5.7) group compared to the non-GDM group (12.6 kg ± 5.2, *p* = 0.001). The fasting BSL, insulin, HOMA-IR, lipid profile, and risk of pre-eclampsia were improved in the GDM group. On the contrary, HbA1C significantly reduced in the non-GDM (5.2% ± 0.3) compared to the GDM group (5.3% ± 0.3, *p* = 0.001) (12). Another intervention trial from the St. Carlos cohort assigned 237 GDM women to MED intervention (*n* = 136) and a control group (*n* = 111) intending to follow them for 3 years post-partum. MED significantly reduced waist circumference [MED = 81 cm (76–87 cm), vs. control = 83 cm (78–93), *p* < 0.05], BMI [MED = 24.7 kg/m^2^ (22.4–27.8), vs. control = 26.7 kg/m^2^ (22.7–30.2), *p* < 0.01]. The glycemic profile of GDM women improved with MED post-delivery with significant reductions in fasting insulin [MED = 2.5 mcUI/ml (2.0–5.6) vs. control = 4.6 mcUI/ml (2.0–7.7), *p* < 0.05], in contrast, fasting blood glucose levels did not improve. Total cholesterol, LDL-cholesterol, triglycerides, apolipoprotein B, and diastolic blood pressure were reduced significantly, further correlating improved healthy fat and nutrition scores (*p* < 0.05) in the MED group (13).

Overall, adherence to MED remarkably improved maternal anthropometry and glycemic profile among women with GDM. Short-term adherence to MED significantly reduced gestational weight gain but did not improve maternal glycemic profile. Similarly, post-partum long-term adherence significantly reduced waist circumference, BMI, and GWG, however, did not improve fasting blood glucose levels (BSL). Nevertheless, long-term adherence lowered the fasting insulin levels as correlated with weight loss. Future studies could focus on comparing MED with a standard diet among GDM women to investigate its benefits over other diets.

### Fetal outcomes

Only one study investigated the fetal outcomes among GDM women following the MED diet. MED intervention reduced birthweight (kg) in GDM (3,126 ± 465) compared to the non-GDM group (3,273 ± 468, *p* = 0.002). Furthermore, a lower proportion of LGA babies were observed in GDM (0.8%) than in the non-GDM group (3.9%, *p* = 0.048). Adherence to the MED diet resulted in fewer NICU admissions in the GDM group. Other fetal outcomes such as SGA, Apgar score (1 & 5 min), hypoglycemia, respiratory distress, and brachial plexus injury did not differ between the groups (12).

Overall, the MED diet positively reduced birth weight, and low incidences of LGA were seen in MED group. Future studies could focus on investigating the effect of MED on long-term fetal outcomes including risk of chronic non-communicable diseases.

## Dietary Approaches to Stop Hypertension (DASH) diet and GDM

The DASH diet is primarily rich in complex carbohydrates, dietary fiber, lean protein, magnesium, potassium, and low energy density. The DASH diet has been well-studied and recommended diet therapy for hypertension, cardiovascular diseases, type II diabetes, and metabolic syndrome. Previous studies have reported the beneficial effects of pre-pregnancy adherence to the DASH diet in reducing the risk of GDM (14, 15). Furthermore, higher adherence to the DASH diet has demonstrated better glycemic control among women with pregestational diabetes (16). However, the effect of the DASH diet after being diagnosed with GDM during the 2nd & 3rd trimesters has been less explored.

### Maternal outcomes

The first-ever, 4-week randomized controlled trial from China investigated the effect of the DASH diet in 33 GDM women. A DASH diet group showed significantly (*p* < 0.05) lower fasting blood glucose levels (reduced by 8.1 mg/dl), serum insulin (reduced to 2.5 Î¼IU/ml), and HOMA-IR scores (reduced to 0.7). Total antioxidant capacity (TAC) (increased to 152.5 mmol/L from 48.1 mmol/L) and total glutathione levels (from 109.5 increased to 155.3 Î¼mol/L, *p* < 0.01) increased significantly in the DASH diet group. This further resulted in forgoing the need for insulin therapy with only 23.5% of participants in the DASH diet group requiring insulin vs. 75% of the participants from the control group (*p* < 0.01). Additionally, only 47.1% of participants from the DASH diet group needed to undergo cesarean section compared to 81.3% of participants from the control group (*p* < 0.01). No effect of DASH intervention was observed for GWG and body mass index (BMI) (17). Another randomized controlled trial from Iran reported similar reductions in fasting blood glucose, serum insulin, and HOMA-IR with the DASH diet. Additionally, TAC increased remarkably in the DASH group post-intervention. However, no significant changes were observed in gestational weight, perhaps because the shorter duration of the trial weight reduction was not achieved to a greater extent. A case-control study by Izadi V et al. (18) compared the effectiveness of the DASH and MED diet on maternal outcomes among 200 GDM and 263 non-GDM women. The results indicated that the higher (third tertile) adherence to DASH and MED diets (*P* = 0.006), lowers the risk of GDM. Importantly, participants with high scores for DASH and MED had significantly low fasting blood glucose, HbA1C, and blood pressure (*P* < 0.05). Furthermore, higher serum HDL-Cholesterol levels (mg/dl) (48.35 ± 9.22 vs. 46.40 ± 9.83) were noted with high adherence to the DASH diet (*P* = 0.004).

In summary, higher adherence to the DASH diet remarkably improved maternal glycemic profile, and increased TAC. Although the DASH diet non-significantly impacted gestational weight in the short term, long-term adherence to the DASH diet may positively prevent gestational weight gain. Further studies are recommended to evaluate the long-term effect of the DASH diet on the future risk of non-communicable diseases among women.

### Fetal outcomes

Only one study evaluated the effect of the DASH diet on fetal outcomes. Higher adherence to the DASH diet by GDM women during 2nd & 3rd trimesters positively reduced birthweight (DASH- 3.2 ± 0.1 vs. control- 3.8 ± 0.1, *p* < 0.0001), head circumference (35.3 ± 0.2 vs. 34.0 ± 0.1, *p* < 0.01), and ponderal index (2.47 Â ± 0.1 vs. 2.9 Â ± 0.1, *p* > 0.0001). However, no changes in the Apgar score were noted (17).

## Plant-based diet (PBD) and GDM

Plant-based diets (PBD) indicate dietary patterns rich in plants and their derivatives excluding animal products. Additionally, terms such as vegan, and vegetarian diets with minimal intake of animal products are also used synonymously to refer to plant-based diets. PBD is rich in antioxidants and has low GI due to high fiber content making it beneficial for diabetic populations. PBDs emerged as a weight-loss diet and are considered an environment-friendly and sustainable food choice. Previous literature has examined the impact of PBD among non-pregnant Type II diabetic individuals (19). Nevertheless, the usefulness of PBD interventions among pregnant women after a GDM diagnosis is less studied.

### Maternal outcomes

Only two studies evaluated the effects of PBD among GDM women. A recent randomized crossover trial (eMom pilot study) compared a plant-based Nordic diet (PBND) with a moderate carb-restricted diet (MCRD- 40% of total energy) in 36 pregnant women with GDM for a short duration. PBND is predominantly a high-fiber diet involving a high intake of complex carbohydrates, legumes, fruits & vegetables, fish, and a low intake of red meats. Glycemic variability (Time in Range-TIR) and control (mean glucose) were recorded using CGM devices. PBND (4.8 mmol/L) significantly (*p* = 0.049) improved mean glucose levels compared to MCRD (4.9 mmol/L) by the end of 3 days. Glycemic variability (TIR) was non-significantly improved with no within-group differences (PBND- 98.9%, MCRD- 98.7%, *p* = 0.727). Contrary to the study's hypothesis, fasting serum insulin [PBND = −0.0 mU/L(−0.8 to 0.8), MCRD = −1.3 mU/L (−2.4 to −0.3), *p* = 0.034] and insulin resistance [PBND = 0.03 (−0.24–0.18), MCRD = −0.37 (−0.68 to −0.06), *p* = 0.030] were improved significantly in the MCRD group. Similarly, the MCRD group showed a remarkable decline in total cholesterol [PBND = −0.0 (−0.2–0.1), MCRD = −0.2 (−0.4 to −0.1), *p* = 0.023], and LDL-cholesterol [PBND = −0.0 (−0.2–0.1), MCRD = −0.2 (−0.3 to −0.1), *p* = 0.030] levels than PBND group (20). PBND was not superior to MCRD with both interventions rendering improvements in maternal glycemic and lipid profiles. However, the duration of the study and sample size were small, therefore results may be non-conclusive. Additionally, it is not surprising to observe improvements in the MCRD group as the beneficial effects of carbohydrate restriction in GDM and Type II diabetes have been documented earlier (21–23).

A case-control study evaluated the effects of PBD among 460 Iranian women [GDM cases (*n*) = 200, Control (*n*) = 260] by analyzing their dietary patterns and calculating the scores for each of the three indices i.e Plant-based Diet Index (PDI), Healthful PDI (hPDI) and Unhealthy PDI (uPDI). PDI indicates the overall consumption of plant-based Iranian foods amongst 18 food groups. These food groups were further categorized as Healthful (Foods rich in complex carbohydrates, fruits, vegetables, legumes, and nuts) and Unhealthful (Sweets, sugar-rich beverages, packed fruit juices, refined carbohydrates, and potatoes) PDI. Women with GDM adhering to plant-based diets (high PDI scores) had significantly low fasting blood glucose (*p* = 0.02), total cholesterol (*p* = 0.05), LDL-C (*p* = 0.04) but high systolic blood pressure (*p* < 0.01) which could be attributed to higher sodium intakes in this group (*p* < 0.001). Relatively, high adherence to unhealthy PDI increased the risk of GDM even after adjusting for energy intake and age (OR_adj_: 1.71; 95% CI: 1.02–2.85) as evidenced by significantly high triglycerides (*p* < 0.001), total cholesterol (*p* = 0.03), and fasting blood glucose (*p* < 0.01) (24).

Overall, both studies showed higher adherence to PBD improved maternal glycemic and lipid profiles however scarce evidence and limitations of available evidence make it difficult to conclude. Additionally, with different definitions of PBDs, the results may not be generalizable.

### Fetal outcomes

There is a severe lack of data exploring the effects of PBD on the health outcomes of fetuses born to women with GDM. We found no direct evidence regarding fetal outcomes among GDM women adhering to plant-based diets, therefore the findings from other studies investigating vegan diets are discussed below. A recent prospective cohort study from Denmark showed an association between maternal adherence to a vegan diet and low birth weight (25). An online retrospective survey of 1,419 women, 4 years postpartum explored the effects of plant-based diets on fetal outcomes. A maternal vegan diet significantly increased the risk of SGA after adjustment for smoking, age, birth week, and GDM. Additionally, low birth weight centiles (42.6 ± 25.9) were seen with adherence to a vegan diet compared to an omnivorous diet (52.5 ± 27.0, *P* < 0.001) (26).

## Low Glycemic Index (GI) diets and GDM

The Glycemic Index (GI) is the potential of food to increase blood sugar levels, specifically, it relates to the carbohydrate type present in foods. Complex carbohydrates have low GI due to their dietary fiber content, whereas simple carbohydrates e.g., glucose, and sucrose have a high GI. Increased consumption of high-GI foods contributes to the development of chronic hyperglycemia. On the other hand, low GI diets have been widely recommended nutrition therapy for the non-pregnant diabetic population (27). Similar positive effects on the metabolic health of women with GDM and their fetuses may be expected.

### Maternal outcomes

A randomized control study involving 140 GDM women subjected to either Low GI (*n* = 66) or standard diabetic diet intervention (*n* = 74) reported significant improvements in glucose levels in both groups (*P* < 0.05) (28). Similarly, no significant differences in low-GI and conventional high-fiber intervention were observed in a pilot follow-up study involving 55 women with GDM. However, low fasting insulin, high insulin sensitivity, HDL-C, and reduction in weight in the low-GI group were observed compared to a conventional high-fiber diet (*p* < 0.05) (29). A Chinese study among 95 GDM women reported a significant (*p* < 0.01) reduction in fasting (mmol/L) (−0·33 vs. −0·02) and 2 h postprandial glucose (mmol/L) (−2·98 vs. −2·51), and total cholesterol (mmol/L) (0·12 vs. 0·23) was noted however, no change in weight gain was seen in a low-GI group (30). Another study by Hernandez TL et al. (31) reported significantly improved insulin sensitivity, fasting blood glucose, inflammation, and free fatty acids in those following the low-GI diet at the 37th week. A cross-over study from Australia also reported similar findings with significant control over glycemic variability and maternal glucose levels (32). A high-fiber diet (LGI) significantly decline in fasting and 2 h postprandial blood glucose (33). Only one study evaluated the effect of low-GI diets in the post-partum (6 years) on the future risk of prediabetes in 281 women with history of GDM. Higher fruit and vegetable intake reduced impaired glucose tolerance significantly [OR = 0.88 (0.81–0.97), *p* < 0.05] (34).

Low GI diets showed a remarkably positive impact on maternal outcomes with better glycemic control in the short term. Further, low GI diets may protect against future risk of prediabetes in women with a history of GDM.

### Fetal outcomes

We identified a total of 5 studies that explored the effect of low-GI diets on fetal outcomes. A pilot follow-up study of 155 infants born to GDM mothers randomized to either a low-GI or high-fiber diet reported no significant differences in length, birth weight, weight for length, and weight gain per day (29). Low-GI diets non-significantly reduced incidences of preterm delivery, macrosomia, and infections in fetuses born to GDM mothers (30). Infant adiposity significantly correlated with insulin resistance *r* = 0.731, *P* < 0.01) and fasting insulin (*r* = 0.697, *P* = 0.01) among GDM mothers at 37th gestational week. Further, the study concluded that infant adiposity may reduce by reducing maternal fasting insulin and insulin resistance through low-GI diets (31). The “GI Baby 3” study from Australia studied health outcomes in infants born to 139 women jeopardized by GDM. The study observed non-significant changes in fetal birth weight, ponderal index, body fat percentage, NICU admissions, SGA, LGA, and macrosomia with low-GI diets (35). A recent prospective cohort study exclusively identified the effect of low-GI diets on fetal outcomes in GDM mothers. The study reported no changes in birthweight and SGA, however, incidences of preterm birth and LGA were significantly lower in the low-GI group (*p* = 0.02). Surprisingly the incidences of preterm birth were higher in quartile 4 of low-GI (6.5%) compared to 1st quartile 5.0%. Moreover, the prevalence of LGA reduced significantly with high adherence to a low-GI diet. Additionally, higher glycemic load in mothers was associated with higher fetal abdominal circumference (36).

Overall, it was noted that maternal adherence to low-GI diets lowered fetal adiposity and the prevalence of LGA. The implications of a low GI diet on adiposity during adulthood among those born to GDM women should be investigated further.

## Ramadan fasting and GDM

Ramadan Fasting (RF), a religious form of intermittent fasting, is followed by Muslim communities worldwide during the holy month of Ramadan. In RF, eating is prohibited for about 12–20 h between sunrise & sunset which varies with geographical location and season. During the eating periods, mostly high carbohydrate and high-fat foods are consumed, however, studies exploring the exact dietary patterns during Ramadan Fasting are lacking (37). All Muslims observe Ramadan fasting except for pregnant, lactating, and menstruating women, older adults, children, and those with acute or chronic diseases. Despite the exemption from RF, pregnant women still opt to fast for different reasons (38). The studies investigating the effect of Ramadan Fasting (RF) on gestational diabetes mellitus (GDM) are negligible. Since RF can be regarded as a form of intermittent fasting, the results may be extrapolated to GDM mothers with various ethnicities but who are following or willing to follow intermittent fasting.

### Maternal outcomes

A few recently published studies among pregnant women with gestational diabetes reported mixed findings. Alasulami S et al. (39) prospectively analyzed 53 GDM women and 17 pregnant women with T2DM who willingly exposed themselves to RF for a median number of 29 days (13.5 h). Fasting blood sugar levels were significantly (*p* = 0.033) improved among both groups. Another study comparing pregnant GDM women (*n* = 57) with pregnant healthy women (*n* = 25) reported statistically significant improvements (*p* = 0.016) in HbA1c levels in both groups wherein HbA1c values reduced as the number of fasting days increased. Fasting for more than 20 days resulted in reduced HbA1c in the GDM group (from 5.7 ± 0.6, reduced to 5.3 ± 0.4) as well as in the non-GDM group (from 5.2 ± 0.5, reduced to 4.6 ± 0.4) (40). Contrary to this, Almogbel et al. (41) reported non-significant changes in blood glucose levels, insulin treatment, preterm delivery, cesarean section, gestational hypertension, and weight gain irrespective of the number of fasting days compared to those who didn't fast. In a retrospective case-control study from UAE, 401 women with GDM or Pre-GDM were grouped as fasted against medical advice (Group A; *n* = 111) and permitted to fast (Group B; *n* = 254). The study compared maternal outcomes such as HbA1c > 9%, insulin use and incidences of hypoglycemia, Diabetic Ketoacidosis (DKA), and acute illness between both groups. A significantly (*p* = 0.0001) higher proportion of participants from Group A had HbA1c > 9% (32% vs. 2%) and high insulin requirement (65% vs. 24%) compared to Group B. The incidences of breaking fast for >1 day were prevalent significantly in (*p* < 0.001) Group A (49%) than in Group B (13%). Hypoglycemia (Group A-63% vs. B- 50%) was the top-most reason for breaking the fast followed by acute illness (Group A-33% vs. B-50%) and DKA (Group A- 4% vs. B- 0%) with significant differences (*p* = 0.039) between the groups (42).

Continuous monitoring of blood glucose in GDM is important, especially during fasting. Recently, a few studies from the UAE have explored the applicability of continuous glucose monitoring (CGM) in GDM women following RF. A study by Hassanein et al. (43) aimed to evaluate the safety of RF and understand glycemic variations as recorded by FreeStyle Libre Flash (FSL)-CGM in 25 women with GDM. The study noted a significant increase in weight (kg) (Pre-RF = 86.8 ± 17.3; Post-RF = 87.9 ± 17.5, *p* = 0.003) and triglyceride levels (mg/dl) (Pre-RF = 225.1 ± 47.2; Post-RF = 267.5 ± 105.1, *p* = 0.014) after RF, whereas, HbA1C (Pre-RF = 5.8 ± 0.5; Post-RF = 5.4 ± 0.5, *p* = <0.001 and % mean above target glucose (Pre-RF = 21.5 ± 7.8; Post-RF = 13.6 ± 7.1, *p* = 0.006) improved remarkably post-RF. The incidences of hypoglycemia increased during RF but were not observed between diet only, diet & metformin, and metformin or insulin groups.

Another study from UAE measured the impact of RF on glycemic parameters in 32 GDM women using CGM devices. The participants from RF only (106 mg/dl ± 9) and RF & Metformin had lower mean glucose levels (99 mg/dl ± 7) compared to the non-fasting group (116 mg/dl ± 21). The severity of postprandial hyperglycemia (>180 mg/dl) was also lower in RF only (23%) and RF & Metformin group (5%) as opposed to the non-fasting group (32%). CGM device readings revealed that hypoglycemia events (at least once a day) were higher in the RF & Metformin group (78%) followed by RF only (60%) and low in the non-fasters (50%) with the majority of incidences (100%) occurring at late fasting hours (16:00–19:00) in a day. Additionally, severe hypoglycemic events (BSL < 50 mg/dl) were high with RF (23%) and low in the RF & Metformin treatment (4%) (44).

In conclusion, RF among GDM women increases the risk of hypoglycemia in addition to glycemic improvements, however, more studies are required to confirm their effectiveness.

### Fetal outcomes

We observed that two studies exploring the effects of RF on fetal outcomes provided much deeper insights than other dietary interventions. A recent case-control study compared the neonatal outcomes among GDM and healthy pregnant women based on the number of fasting days (<11 days, 11–20 days, <20 days). The results indicated a non-significant effect of RF on neonatal outcomes except that head circumference differed significantly in both GDM and non-GDM groups after RF. Additionally, head circumference (cm) increased as the number of fasting days increased in the GDM group (32.6 ± 0.9, 33.5 ± 2.1, 33.4 ± 1.3, *p* = 0.055). In contrast, the non-GDM group showed a distinct pattern wherein head circumference increased significantly (*p* = 0.038) in those who fasted for 11–20 days (37.2 cm ± 7.8) with a reduction in those fasting for more than 20 days (32.5 ± 1.4) compared to <11 days fasting (33.3 ± 0.6) (40). Increased head circumference in the GDM group indicates newborn macrosomia, an anticipated risk factor in gestational diabetes (45), therefore whether RF has any impact on macrosomia warrants further exploration.

Another retrospective cohort study among 345 GDM women following RF, noted certain neonatal complications. The number of fasting days, duration of fast/day, and trimester during fasting significantly affected the neonatal complications. For e.g., neonatal hyperbilirubinemia significantly (*p* = 0.004) increased as fasting days increased (1–10 days = 7.1%, 11–20 days = 13%, and 21–30 days = 22.8%) compared to the non-fasting group (6.8%). The fasting duration of 12–13 h (25.8%) and 14–15 h (33.8%) significantly (*p* ≤ 0.05) increased incidences of neonatal hyperbilirubinemia compared to the non-fasters (6.8%). On the contrary, the prevalence of neonatal hypoglycemia declined significantly as the number of fasting days (1–10 days 26.7%, 11–20 days 0%, 21–30 days 10.4%; *p* = 0.05) and fasting duration increased (12–13 h = 9.7%, 13–14 h = 11.3%, >14–15 h = 16.3%, >15–16 h = 7.2%; Z*_trend_ p* = 0.06) as opposed to the non-fasting group with high neonatal hypoglycemia rates (22.7%). The mean birthweight increased as the duration of fasting (12–13 h = 3,107 gms ± 521, 13–14 h = 3,197 ± 598, >14–15 h = 3,265 ± 488, >15–16 h = 3,347 ± 584) increased significantly (*p_trend_* = 0.02) compared to the non-fasters (3,140 ± 619). The prevalence of SGA was high (12.8%) in the non-fasting group compared to the fasting group with significant (*p* = 0.03) differences in fasting duration (12–13 h = 3.2%, 13–14 h = 12%, >14–15 h = 0, >15–16 h = 3.6%) but not in the number of fasting days (1–10 days- 0%, 11–20 days- 13%, 21–30 days- 5.6%; Z*_trend_ p* = 0.1). Furthermore, the exposure to fasting during later trimesters (2nd and 3rd trimesters) significantly increased the birthweight (1st trimester- 3,163 gms ± 603, 2nd- 3,272 ± 523, 3rd-3,851 ± 497; *p* = 0.03) and prevalence of neonatal hyperbilirubinemia (1st trimester- 16.4%, 2nd- 23.3%, 3rd- 25%; *p* = 0.02), in contrast to the reduced incidences of neonatal hypoglycemia (1st trimester- 10.9%, 2nd- 8.3%, 3rd- 8.9%; *p* = 0.05) (41).

Although maternal adherence to RF decreased the prevalence of neonatal hypoglycemia, the longer fasting duration led to increased birth weight, head circumference, macrosomia, and higher incidences of neonatal hyperbilirubinemia. Overall, the undesirable effects of maternal RF during 2nd & 3rd trimesters were observed among fetuses born to GDM women.

## Calorie restricted diets and GDM

A calorie-restricted (CR) diet focuses on limiting energy without malnutrition. CR was originally recommended to promote weight loss and has been studied extensively among overweight, and obese individuals (46). A recent systematic review concluded that CR diets may promote remission of diabetes and lower the cardiometabolic risk in Type II diabetic individuals (47). Although CR diets emphasize energy restriction without malnutrition, CR during a critical stage like pregnancy may be counter-intuitive and may even have adverse maternal and fetal outcomes. On the contrary, CR diets in GDM may also result in weight loss promoting improved insulin sensitivity and decreased insulin resistance thereby reducing the GDM-associated adverse outcomes. There is no standardized range for the amounts of calories restricted per day, rather it is determined based on individual requirements. Therefore, there is a large variation in studies discussed below regarding the ideal number of calories to be restricted.

### Maternal outcomes

The effect of CR diets in women with GDM remains largely unknown with only three studies having attempted to investigate. A recent TIMER (The gestational diabetes Mellitus Energy Restriction) study compared a low-energy (LED- 1,800 kcal) vs. a very low-energy diet (VLED- 1,600 kcal) intervention in 43 pregnant women with GDM. The LED group (11.4 ± 5.0) had higher total gestational weight gain (GWG) whereas, the VLED group experienced a weight loss (6.8 ± 7.6). A remarkable (*p* = 0.02) rise in MUAC in the VLED group (31.5 ± 3.18) was observed compared to the LED group (28.3 ± 3.92). Other maternal outcomes such as gestation age (GA), C-section delivery, and insulin use, were non-significantly different in both groups. The majority had GA between >37, <42 weeks (LED-100%, VLED- 93.3%, *P* = 0.48). The proportion of C-section delivery was non-significantly (*p* = 0.35) higher in VLED (66.7%) as opposed to the LED group (50%). The VLED group required insulin relatively early (22 ± 8 GW) compared to LED (25 ± 7 GW). Additionally, more insulin units were required at labor in VLED (26.8 ± 13.6 units, *p* = 0.36) than in LED (18.9 ± 13.7 units, *p* = 0.27). Another case-control study among 16 GDM women investigated the impact of moderate energy restriction (1,200 kcal/day). CR resulted in significant weight loss per week (mean weight loss 0.4 ± 0.4 kg) (*p* < 0.001). Overall weight loss was seen in CR (1.6 ± 1.7 kg) vs. in control group (1.4 ± 1.2 kg). A median liver triacylglycerol also reduced from 3.7%–1.8% significantly in the CR group (*p* = 0.004) post-intervention. Surprisingly, despite significant weight loss, there was no change in fasting and post-prandial blood glucose, fasting insulin, HOMA2 index, and lipid profile in both groups. This was attributed to the small sample size and inability to control for certain factors (48).

Only one study by Gray KL et al. (49) investigated the long-term impact of calorie restriction in women with a GDM history. A recent 12-month RCT compared the effects of very low-calorie, 2-day Intermittent energy restriction (IER- 500 kcal, *n* = 61) with Continuous moderate energy restriction (CER- 1,500 kcal, *n* = 60). A statistically significant (*P* < 0.001) weight loss over time was observed and an average weight loss of 4.3 ± 5.5% compared to baseline was noted at the end of the 12-month trial with no differences (*p* = 0.2) within the two groups. Maternal glycemic profile (fasting blood glucose, HbAlc, serum insulin levels, and HOMA-IR) improved significantly (*P* < 0.001) over 12 months in both groups but no change in postprandial glucose levels was observed. Additionally, weight loss significantly (*p* = 0.03) correlated with reductions in HbA1c, fasting insulin (*r*^2^ = 0.07) and change in HOMA-IR (*r*^2^ = 0.08) in both groups.

In summary, short and long-term CR resulted in weight loss in women with GDM. Despite the weight loss, short-term CR did not improve the glycemic profile. In contrast, long-term CR postpartum in women with a history of GDM significantly improved HbA1c, fasting insulin, and HOMA-IR. Different maternal outcomes and a few limitations affect the definite conclusion. Nevertheless, long-term CR in women with a history of GDM has the potential to mitigate the future risk of non-communicable diseases including type II diabetes, however, more evidence is warranted to support this notion.

### Fetal outcomes

We found only two studies investigating the impact of CR on fetal outcomes. The TIMER study reported that CR non-significantly influenced fetal outcomes such as birthweight, premature birth, SGA, LGA, and APGAR score at 1 and 5 min. The birthweight was lower in VLED than in the LED group (3,114 ± 400 vs. 3,011 ± 333, *p* = 0.44). CR increased maternal urinary ketone production; however, the results were non-significant. Nevertheless, it is worth reiterating that increased maternal urinary ketones have been associated with adverse fetal outcomes (50, 61). Therefore, until strong evidence is available, CR among GDM women may not be advisable. In contrast, another study reported LED (3,360 ± 277) did not reduce birthweight as opposed to the control diet (3,361 ± 398, *p* = 0.99). Another fetal outcome assessed in this study was special unit admissions which did not differ between both groups (LED-1, Control-2, *p* = 0.84) (48).

Both short-duration studies showed no significant impact of CR on fetal anthropometric measures such as birth weight. Future studies are recommended to assess the effect of short and long-term CR on fetal outcomes such as macrosomia, SGA, LGA, neonatal glycemic profile, and risk of hyperbilirubinemia.

## Low-carbohydrate (low-carb) diets and GDM

Carbohydrates are a prime energy substrate and the only macronutrient that directly influences blood glucose levels. Consumption of carbohydrate-rich diets in pregnancy with GDM could potentially increase hyperglycemia and adversely affect maternal and fetal health. Low-carb diets ameliorate the glycemic profiles of non-pregnant diabetic populations (23), but their effect on the glycemic profiles among the GDM population is understudied.

### Maternal outcomes

A case-control study from China treated women with (cases-152) and without GDM (control-113) to low-carb and control diets in addition to regular medical management. A low-carb diet significantly reduced fasting (*p* = 0.020) and post-prandial blood sugar levels (*p* = 0.049) among those not treated with insulin compared to insulin-treated women. The need for insulin was significantly (*p* < 0.001) delayed by 3.47 weeks among the low-carb diet group (33.59 ± 3.45) compared to the control group (29.21 ± 4.07). However, no significant changes were observed in gestational weight gain, hemoglobin, and lipid profiles of the participants in both groups (51). In contrast, a randomized control trial assigned 46 GDM women to follow a low-carb diet (135 gms/day) reported non-significant changes in fasting (low-carb = 4.9 ± 0.1 vs. standard 4.9 ± 0.1, *p* = 0.88) and post-prandial (low-carb = 6.5 ± 0.1 vs. standard 6.2 ± 0.1, *p* = 0.17) blood sugar levels; whereas no changes in gestational weight gain were seen post-intervention. When comparing the mode of deliveries, no significant changes were observed except for reductions in elective cesarean delivery among a low-carb (12.5%) compared to a standard diet (38.1%) (*p* < 0.05) (52). A similar study compared a high-carb diet (*n* = 6) with a low-carb diet (*n* = 6) among pregnant women with GDM measured glycemic variability and control using CGM devices and other maternal outcomes such as HOMA-IR, lipid profile, and C-reactive proteins were assessed. The glycemic variability measured as the mean amplitude of glucose excursions (MAGE) significantly (*p* = 0.004) reduced (High-carb = 1.8%, Low-carb = 0.5%), in contrast, non-significant (*p* = 0.08) improvements in time in range (TIR-%) were observed in a low-carb group (High carb = 8.7%, Low-carb = 3.7%). Contrary to the hypothesis, a high-carb diet significantly declined the fasting (mmol/L) (High-carb = 4.62, Low-carb = 5.07, *p* = 0.007) and mean glucose (mmol/L) (High-carb = 4.9, Low-carb = 5.2, *p* = 0.02) compared to a low-carb diet post-intervention. A similar trend was observed between both groups for insulin resistance (High-carb = −0.214, *p* = 0.02, Low-carb = −0.107, *p* = 0.68). These improvements in glycemic control and insulin resistance in a high-carb group may be attributed to non-significant differences in mean consumption of carbohydrates (high-carb = 222 g vs. low-carb = 215 g, *p* = 0.16) and dietary fiber (High-carb = 38.79 g vs. Low-carb = 39.50, *p* = 0.68) (21).

A randomized controlled trial administered a low-carb diet (35%–40% of total energy) to GDM women from urban (Site A) and semi-urban (Site B) hospital sites. The study reported no changes in maternal outcomes for gestational weight change, fasting, and postprandial blood glucose levels, insulin need was observed in the low-carb group. On the contrary, significant changes in 2-hr post-prandial glucose levels were seen when compared based on study locations (Site B- 100.59 ± 7.30 vs. Site A- 116.30 ± 15.13, *p* < 0.01) (53). One of the possibilities for these variations could be due to significantly higher intakes of protein (100.17 g) and fats (89 g) at Site B in contrast to Site A (protein- 82.3 g, fats- 65 g, *p* < 0.01 for both nutrients). In contrast, a low-carb diet increased incidences of C-section delivery (low-carb = 33.8% vs. control = 26.7%) and moderate to high ketonuria (low-carb = 22.9% vs. control = 20.6%). A Low-carb diet significantly resulted in reduced gestational weight gain (low-carb = 1.4 ± 2.0 vs. control = 2.3 ± 2.0, *p* = 0.017) and low prevalence of maternal hypertension, without any significant changes in maternal fasting BSL, 1 and 2 h postprandial glucose, and insulin requirement (54).

Only one prospective cohort trial explored the risk of T2DM in the future among 722 women with GDM history with adherence to a low-carb diet. Overall, a low-carb diet significantly increases the risk of T2DM in later life [2.13 (1.65–2.76), *p* < 0.001]. Interestingly, this study noted that low-carb, vegetable-based protein and fats (1.29 (1.00–1.67) (*P* = 0.14) reduced the odds of T2DM; whereas, low-carb, animal-based protein and fats [2.18 (1.68–2.83), *p* < 0.001] significantly increased the risk of T2DM in future (55).

In summary, the present evidence showed that a short-term low-carb diet does not significantly improve the glycemic profile and certain maternal outcomes. In contrast to the notion, adherence to a low-carb diet by GDM women does not decrease the risk of T2DM. Importantly, energy supplemented through animal proteins and fats in low-carb diets may aggravate the risk of T2DM in women with GDM.

### Fetal outcomes

Only three studies followed GDM women adhering to the low-carb diet to explore its impact on fetal outcomes. A randomized controlled trial noted no impact on fetal outcomes such as birthweight (gm) (low-carb = 3,125 ± 101, control diet = 3,278 ± 79, *p* = 0.25), SGA (low-carb = 25%, control diet = 14.3%, *p* = 0.25), LGA (low-carb = 0%, control diet = 4.8%, *p* = 0.28), and macrosomia (low-carb = 4.2%, control diet = 4.8%, *p* = 0.55). Increased muscle mass (low-carb = 92.8% ± 2.2, control diet = 89.9 ± 1.0, *p* = 0.23) and reduced fat mass (low-carb = 7.2% ± 2.2, control diet = 10.1% ± 1.0, *p* = 0.23) were noted in the low-carb group however, the results were non-significant (52). Perhaps these changes may be due to the small size and non-inclusion of other maternal variables which might cause these improvements for e.g., physical activity/exercise because maternal dietary data do not explain improvements in fat and muscle mass otherwise.

The US-based study reported no significant reduction in hypoglycemic events, SGA, LGA, and macrosomia in a low-carb diet (54). Another study also observed a non-significant increase in birthweight (Low-carb = 3,409.53 g ± 527.91, Standard = 3,377.28 g ± 589.91, *p* = 0.81), head circumference (Low-carb = 35.09 cm ± 3.80, Standard = 33.95 cm ± 1.77, *p* = 0.13), abdominal girth (Low-carb = 31.78 ± 2.83, Standard = 31.56 ± 3.17, *p* = 0.77), incidence of shoulder dystocia (Low-carb = 2.9%, Standard = 0%, *p* = 0.25) and hospitalization (Low-carb = 20.6%, Standard = 12.5%, *p* = 0.38) (56).

The current literature concerning the effects of a low-carb diet on fetal outcomes presented mixed findings. One study reported improvements in neonatal anthropometry whereas another showed undesirable changes. The shorter study duration and small sample sizes make it difficult to generalize the findings highlighting the need for future studies.

## High protein diets (HPD) and GDM

Protein is one of the crucial macronutrients for optimum growth and development of the fetus. Protein synthesis increases markedly during the latter half of the pregnancy (2nd & 3rd trimester) besides increased insulin resistance. High protein diets (HPD) that emerged as popular weight loss strategies have now been widely recommended to attenuate blood glucose levels among the non-pregnant diabetic population (57). Considering the increased protein requirements alongside increased insulin resistance as evident in GDM, recommending HPD may have synergistic benefits, thereby improving obstetrics and fetal outcomes. However, the evidence supporting the benefits of adhering to HPD after a GDM diagnosis is lacking.

### Maternal outcomes

A recent 3-day randomized controlled trial compared HPD (protein = 30%; CHO = 35%) with a low-protein diet (LPD) (protein = 15%; CHO = 50%) in 12 pregnant women with GDM, reported significant reductions (*p* ≤ 0.05) in glucose and insulin area under curve (iAUC) and low postprandial mean blood glucose levels (56). Although statistically significant improvements were seen in glycemic and insulinemic response to HPD, the results should be interpreted cautiously due to the intervention's shorter duration (2 days). Additionally, the proportion of carbohydrates in both diets (HP = 35% vs. LP = 50%) was lower and may have also contributed to improved glycemic and insulinemic response. Another randomized cross-over trial reported similar improvements in glycemic profiles. They compared a high protein Soy-based diet (*n* = 32) with a high fiber diet (*n* = 30) among women with GDM. The study reported significant reductions in post-prandial blood glucose levels (*p* ≤ 0.05) and the need for insulin (*p* = 0.05) in the soy-based diet compared to the high-fiber diet after two weeks of intervention. Surprisingly, this difference became insignificant by the end of the trial with no changes in post-prandial blood glucose levels, however, the insulin requirement significantly reduced in the soy-based diet (18.75%) as opposed to high fiber diet (50%, *p* = 0.015) post-intervention. Furthermore, no significant changes in other maternal outcomes such as gestational weight gain, cesarean delivery, fasting blood sugar levels, and HbA1c were observed (58). One of the reasons for non-significant maternal outcomes could be attributed to the low glycemic index of high-fiber diets. A similar study explored the impact of a soy-based high-protein diet (*n* = 34) with a standard diet as a control (*n* = 34) among pregnant women with GDM. The study revealed significant improvements in fasting blood sugar levels (Soy = −12.7 ± 13.2 mg/dl vs. standard = +1.4 ± 11.6, *P* < .001), serum insulin (Soy = −0.9 ± 10.0 *μ*IU/ml vs. standard = +5.0 ± 11.6, *P* = .02), HOMA-IR (Soy = −0.8 ± 2.2 vs. control = +1.2 ± 2.7 *P* = .002), QUICKI (quantitative insulin sensitivity check index) (Soy = +0.01 ± 0.03 vs. control = −0.007 ± 0.02, *P* = .004) and lipid profile in the high protein group post 6 weeks intervention with standard diet as a comparator (59).

Overall, three interventional studies showed improved maternal glycemic profiles in the short term; however, long-term effects must be evaluated. Additionally, two of the studies compared the effects of plant-based protein (Soy), therefore whether the plant-based proteins are superior in improving glycemic profile compared to animal proteins requires further evaluation.

### Fetal outcomes

Only two studies evaluated the implications of HPDs in fetuses born to GDM mothers. A randomized controlled trial reported that the Soy-protein diet neither improved nor worsened certain fetal outcomes such as preterm delivery, length, macrosomia, head circumference, birth weight, APGAR score at 1/5 min, and neonatal hypoglycemia. Nevertheless, a soy-protein diet significantly reduced neonatal hyperbilirubinemia (Soy = 8.8%, Control = 32.4%, *p* = 0.01) and hospitalization (Soy = 2.9%, Control = 20.6%, *p* = 0.02) (59).

An Indian study reported non-significant changes in incidences of LGA, shoulder dystocia, neonatal hypoglycemia, and TSH levels in both Soy and high-fiber diets. Importantly, neonatal birth weight was significantly lower in the soy-protein (2.86 ± 3.07) compared to the high-fiber group (3.07 ± 0.39) (58), however, it does not indicate any abnormality since the mean birth weights of both groups are within normal ranges. Only 1 study evaluated the future implications of high protein intakes on the metabolic health of children at the age of 9–16 years born to GDM mothers. Protein intake was not associated with changes in fasting insulin and HOMA-IR among children. High protein intake was non-significantly associated with a modest increase in abdominal obesity, however, it did not result in overweight/obesity. Significant differences between the maternal consumption of animal proteins and the impact on metabolic outcomes among offspring in both GDM and non-GDM exposed groups were observed. Interestingly, intakes of dairy and white meat increased the adiposity in the offspring of both groups, whereas, increased insulin resistance was observed with red & processed meat consumption in the offspring of the control group. Gender differences were observed where male offspring had higher insulin resistance than females (60).

In summary, two studies evaluated the short-term effect of maternal protein intakes on different fetal outcomes. High-protein diets neither improved nor worsened fetal metabolic health, except for positive changes such as reduced neonatal hyperbilirubinemia, birth weight, and hospitalization. Higher protein intake resulted in a modest increase in abdominal obesity over the long term in the offspring born to GDM mothers; however, the results were non-significant and require validation from future studies.

## Conclusion

Gestational diabetes mellitus complicates the pregnancy and adversely affects materno-fetal outcomes in both the short-term and future. Very little is known regarding the effects of dietary interventions on maternal and fetal outcomes when followed during 2nd and 3rd trimester after being diagnosed with GDM. Hence, this review focused on pregnant women following various dietary interventions upon receiving a diagnosis of GDM. Eight popular dietary interventions were evaluated and most studies have primarily investigated short-term maternal and fetal outcomes, with very few exploring potential future health implications in both mother and child. Our findings underscore that adherence to Mediterranean, DASH, and low-GI diets during 2nd & 3rd trimesters after being diagnosed with GDM positively affected maternal and fetal outcomes, whereas more studies are warranted to confirm the effects of Ramadan Fasting, Calorie restriction, Low-carbohydrate, and high protein diets Although the beneficial effects of some dietary interventions were noted, the potential sources of bias such as small sample size, short intervention duration, adherence to interventions, differences in outcomes assessed, and inconsistencies in dietary interventions/compositions of meals (For example., studies examining low-carb or High protein diets determined individualized nutrient requirements rather than reference requirements standard for all subjects which may have affected the results) cannot be overlooked while interpreting these findings. Further cultural differences and regional dietary habits may have also affected the results. There is an urgent need for better-quality evidence to confirm the implications of these dietary interventions in women with GDM. Future studies are recommended to examine the effects of dietary interventions on long-term materno-fetal outcomes. Additionally, the critical comparison of these dietary interventions will reveal the best possible dietary solution for preventing the ramifications of GDM on the prospective health of mother and child.
