# Supplementary material for: Maternal and fetal outcomes in gestational diabetes mellitus: a narrative review of dietary interventions
Source: Front Glob Womens Health. 2025 Feb 26;6:1510260. doi: 10.3389/fgwh.2025.1510260 (PMC11897047; doi:10.3389/fgwh.2025.1510260)
Supplement: Supplementary file 1 [file Table1.docx]

**Supplementary table 1: Inclusion & Exclusion Criterion (PICOT framework)**

|  | **Inclusion** | **Exclusion** |
| --- | --- | --- |
| Population | -Women diagnosed with Gestational diabetes mellitus (GDM) in 2^nd^ or 3^rd^ trimester  -Women with a history of GDM  -Women with pre-GDM (Type I or Type II DM) | -Healthy pregnant women  -Animal studies |
| Intervention | 8 Popular dietary interventions such as Mediterranean, DASH, Plant-based, Low-GI, Ramadan Fasting/ Intermittent Fasting, Calorie restriction, Low-carbohydrate, High protein diets followed immediately after receiving a diagnosis of GDM (2^nd^ & 3^rd^ trimesters) were reviewed | Pre-conceptional/ pre-natal Dietary interventions that explored the risk of GDM were excluded. |
| Control | A Standard balanced diet as a control | - |
| Outcome | **Maternal Outcomes:**  -Short-term outcomes such as gestational age, gestational weight gain, waist circumference, BMI, Fasting & Post-prandial blood glucose levels, HbA1c, HOMA-IR, risk of hypoglycemia, insulin requirement, Lipid profile- total cholesterol and LDL-cholesterol, cesarean delivery, and preeclampsia  -Long-term outcomes such as insulin resistance, Prediabetes (Impaired Blood Glucose, Impaired Fasting Glucose), Type II diabetes, Obesity, Cardiovascular diseases  **Fetal Outcomes:**  -Short-term outcomes such as birth weight, stillbirth, preterm birth, macrosomia, neonatal death, shoulder dystocia, hypoglycemia, hyperglycemia, hyperbilirubinemia, risk of hospitalization/ NICU admission  - Long-term outcomes such as adiposity, childhood obesity, insulin resistance, high blood pressure, metabolic syndrome, early onset of cardiovascular diseases | - |
| Timeline | Articles published between the year 2013- April 2024 | - |
| *Other than the PICOT framework we excluded articles for reasons such as 1. No full text available, 2. published in a language other than English, 3. Articles exploring outcomes of interest out of review’s scope | | |

**Mediterranean Diet**

| **Author & Year** | **Country** | **Study design** | **Sample size** | **Study population** | **Dietary intervention** | **Assessed outcomes of interest** | **Advantages** | **Disadvantages** |
| --- | --- | --- | --- | --- | --- | --- | --- | --- |
| Garcia de la Torre, et al., 2019 | USA | Prospective unicentric, interventional study  (St Carlos study) | 932 | GDM and non-GDM women | Nutrition education for MD diet | 1. Anthropometry  2. Glycemic profile  3. Lipid profile  4. Pre-eclampsia  6. Type of delivery | 1. Excessive weight gain compared to AWG was significantly lower in GDM group RR 0.91 (0.86–0.96), *p* < 0.001 at 36-38^th^ GW,  2. No differences in type of delivery  Post delivery (12-14 weeks)  1. Reduced GWG in GDM (10.0 ± 5.7), compared to non-GDM (12.6 ± 5.2, p=0.001) | 1. No improvements in FBSL, F-insulin, HOMA-IR  2. No improvements in lipid profile and pre-eclampsia risk  3. HbA1c improved in non-GDM group significantly (5.2 ± 0.3) than GDM (5.3 ± 0.3, p=0.001) |
| Perez-Ferre N et al., 2015 | USA | RCT  (St Carlos study) | 237 (136-intervention, 111-control) | GDM women (24^th^ 28^th^ GW) | Nutrition education for MD diet + physical exercise with 3 year follow-up post-delivery | 1. Anthropometry  2. Glycemic profile  3. Lipid profile | 1. Reduced BMI 26.7 (22.7e30.2) vs 24.7 (22.4e27.8), p<0.01  2. reduced WC 83 (78e93) vs 81 (76e87), p<0.05  3. Reduced fasting insulin 4.6 (2.0e7.7) vs 2.5 (2.0e5.6), p<0.05  4. Reduced DBP, Total cholesterol, LDL, triglycerides, apolipoprotein B,  5. improved healthy fat score, nutrition score | 1. No improvements in Fasting BSL |
| **Fetal outcomes** | | | | | | | | |
| Garcia de la Torre, et al., 2019 | USA | Prospective unicentric, interventional study | 932 | GDM and non-GDM women | Nutrition education for MD diet | 1. Anthropometry  2. NICU admissions  3. Apgar score  4. Hypoglycemia  5. Hyperbilirubinemia  6. Respiratory distress  7. Brachial plexus injury | 1. Reduced birth weight (3126 ± 465, p=0.002 vs non-GDM= 3273 ± 468)  2. Low proportion of LGA (GDM- 1 (0.8), non-GDM- 31 (3.9), p=0.048)  3. lesser NICU admissions in GDM group compared to non-GDM | 1. Non-significant differences in SGA, Apgar score, hypoglycemia, respiratory distress, brachial plexus injury |

**DASH Diet**

| **Author & Year** | **Country** | **Study design** | **Sample size** | **Study population** | **Dietary intervention** | **Assessed outcomes of interest** | **Advantages** | **Disadvantages** |
| --- | --- | --- | --- | --- | --- | --- | --- | --- |
| Izadi V et al., 2016 | Iran | Case-control study | 263 control, 200 cases | GDM and non-GDM women | DASH vs MD diet | 1. Risk of GDM with adherence  2. Glycemic profile  3. Lipid profile | 1. Lowered risk of GDM with high adherence (3^rd^ tertile) to DASH (OR: 0.29 (0.17-0.48); P = 0.006) and MD (OR: 0.20 (0.50-0.70); P = 0.006)  2. Reduced FBS, blood pressure, and HbA1C (P<0.05).  3. Increased HDL-cholesterol in the DASH group (3^rd^ tertile-48.35±9.22 vs. 1^st^ tertile- 46.40±9.83 mg/dl, P=0.004). | 1. Reduced LDL-cholesterol in MD diet (3^rd^ tertile- 166.39±34.60 vs. 1^st^ tertile-179.76±34.51 mg/dl, P=0.03) |
| Yao J et al., 2015 | China | RCT | 16 control, 17 DASH diet | GDM women | DASH vs control diet | 1. Anthropometry  2. Gestational age  3. C-section delivery  4. Insulin requirement  5. Glycemic profile  6. Polyhydramnios  7. TAC and GSH levels | 1. Significant reduction in c-section delivery (8 (47.1)) vs 13 (81.3), p<0.01  2. Reduced insulin requirement 23.5% vs 75%, p<0.0001  3. Reduced macrosomia prevalence (5.9% vs 37.5%, p<0.001)  4. Reduction in fasting BSL (-8.1 Â± 2.3 vs 3.7 Â± 4.7, p=0.01), Insulin- (-2.5 Â± 1.8, vs 4.3 Â± 2.4, p=0.02) and HOMA-IR (-0.7 Â± 0.5, vs 1.2 Â± 0.8, p=0.02)  5. TAC (48.1 Â± 14.8, vs -152.5 Â± 41.5, p<0.0001) and GSH levels (109.5 Â± 39.7 vs -155.3 Â± 46.8, p<0.0001) improved in DASH | 1. Higher GA in DASH group (38.5 Â± 1.3) vs control= 37.9 Â± 1.5, p=0.53  2. Non-significant reduction in incidences of polyhydramnios |
| Asemi Z et al., 2013 | Iran | RCT | Control (n=16), DASH (n=16) | GDM women | DASH vs Control diet | 1.Anthropometry  2.Glycemic profile  3. Insulin resistance  4.Oxidative stress | 1. Reduced BSL, insulin and HOMA-IR significantly improved in DASH diet  2. Total antioxidant capacity significantly increased in DASH diet | 1.Non-significant reduction in gestational weight in DASH diet group |
| **Fetal outcomes** | | | | | | | | |
| Yao J et al., 2015 | China | RCT | 16 control, 17 DASH diet | GDM women | DASH vs control diet | 1.Anthropometry  2.Macrosomia  3. Ponderal index  4. Apgar score | 1.Reduced birthweight 3.2± 0.1 vs 3.8± 0.1, p<0.0001  2. Reduced head circumference (35.3± 0.2 vs 34.0± 0.1, p<0.01)  3. Rediuced ponderal index (2.47 Â± 0.1 vs 2.9 Â± 0.1, p>0.0001) | 1. No change in apgar score |

**Plant-based Diet (Maternal outcomes)**

| **Author & Year** | **Country** | **Study design** | **Sample size** | **Study population** | **Dietary intervention** | **Assessed outcomes of interest** | **Advantages** | **Disadvantages** |
| --- | --- | --- | --- | --- | --- | --- | --- | --- |
| Zamani B. et al., 2019 | Iran | Case-control | 460, 200- cases, 260- controls | 25-28th GW with GDM, no T1/T2DM | None, 3 food records of 24 hrs to calculate PDI | 1. Maternal blood glucose  2. Maternal lipid profile | 1. Reduced risk of GDM (OR_a_: 0.47; 95% CI: 0.28–0.78) with high PDI scores  2. High unhealthy PDI scores increased odds of GDM (OR_a_: 1.71; 95% CI: 1.02–2.85)  2. Reduced FBG (P = 0.02)  3. Reduced TC (P = 0.05), LDL (P = 0.04), but higher SBP (P = <0.01). | 1. Case-control design disabled understanding of causal relation  2. No other outcomes were assessed  3. Use of three, 24-hr food records  4. Recall/reporting bias |
| Markussen, L.T. et al., 2023 | Finland | Randomized cross-over parallel pilot study | 36 | GDM women (24-28^th^ GW) | 3-day plant-based Nordic diet (PBND) vs moderate carbohydrate restriction (MCRD) intervention with 3-day washout periods | 1. Maternal blood glucose, and insulin resistance  2. TIR using CGM  3. Maternal lipid profile | 1. Small yet significant reduction in 3-day mean glucose in PBND compared to MCRD (4.8 SD 0.5 vs. 4.9 SD 0.5 mmol/L, *p* = 0.049)  2. Reduced fasting insulin, HOMA-IR, TC, and LDL-C in the MCRD diet but not in HND  3. Overall, both diet types reduced mean blood glucose | 1. Small sample size  2. Short study duration |

**Low-GI Diet**

| **Author & Year** | **Country** | **Study design** | **Sample size** | **Study population** | **Dietary intervention** | **Assessed outcomes of interest** | **Advantages** | **Disadvantages** |
| --- | --- | --- | --- | --- | --- | --- | --- | --- |
| Hu Z et al., 2014 | China | RCT | 140 | GDM | Low GI vs standard diabetic diet | Glycemic profile | Significant reductions in fasting and PP BSL in low GI group | - |
| Louie Yu et al., 2015 | Australia | Follow-up RCT | 99 | GDM women followed up till 3 months post-partum | Low GI vs High fiber diet | -Glycemic profile  -Anthropometry | No adverse effects | -No significant improvements in glycemic profile and insulin levels  -No changes in GWG, waist circumference and post-partum weight-loss |
| Ma WJ et al., 2015 | China | RCT | 95 | GDM women | Low-GI vs control diet | -Glycemic profile  -Anthropometry  -Lipid profile | -Fasting and 2-hr PP BSL improved significantly (p<0.05)  -Serum total cholesterol and triglycerides reduced whereas HDL-cholesterol increased (p=0.01) | -No change in GWG |
| Hernandez TL et al., 2016 | Canada | 12-week RCT | 12 | GDM women | High-complex carb-low fat vs standard diet | -Glycemic profile | - | -Fasting BSL increased  - No improvements in insulin sensitivity |
| Kizirian NV et al., 2017 | Australia | Randomized cross-over trial | 17 | GDM women | Low GI vs High GI | -Glycemic control and variability using CGM device | -Highest glucose value was significantly lower  -Mean amplitude of glycemic excursions (MAGE), incremental area under curve (iAUC) and time in target were significantly lower |  |
| Lv Shaofang et al., 2019 | China | RCT | 134 | GDM women | Low GI vs control diet | -Glycemic profile  -Premature delivery | - Fasting and PP BSL significantly improved  -Lower proportions of preterm delivery |  |
| Mercier R et al., 2019 | Canada | Cross-sectional study | 281 | Women with a history of GDM (6 years post partum) | Adherence to fruit & vegetable rich dietary pattern | -Glycemic profile  -Anthropometry  -Risk of IGT | -Significant improvements in HbA1C, fasting and PP BSL, fasting insulin & HOMA-IR were observed.  -Risk of prediabetes was significantly lower |  |
| **Fetal outcomes** | | | | | | | | |
| Louie Yu et al., 2015 | Australia | Follow-up RCT | 99 | GDM women followed up till 3 months post-partum | Low GI vs High fiber diet | Anthropometry | No adverse effects | -No changes in weight for age and height for age |
| Ma WJ et al., 2015 | China | RCT | 95 | GDM | Low-GI vs control diet | Anthropometry | No adverse outcomes | No changes in birth weight and incidences of preterm birth, macrosomia was non-significantly lower |
| Hernandez TL et al., 2016 | Canada | 12-week RCT | 12 | GDM women | High-complex carb-low fat vs standard diet | -Anthropometry | -Fetal adiposity significantly reduced with reductions in maternal fasting insulin and insulin resistance | - |
| Markovic TP et al., 2016 | Australia | RCT | 139 | GDM women | Low GI vs control diet | -Anthropometry | No adverse outcomes | No significant changes in birthweight, ponderal index, body fat percentage, SGA, LGA and macrosomia |
| Wahab R et al., 2021 | Netherlands | Prospective Cohort Study | 3471 | Women with or without GDM | No intervention, Low GI diet pattern was assessed | -Anthropometry | -Significantly lower incidences of preterm birth and LGA | No effects on birthweight and SGA |

**Ramadan Fasting**

| **Author & Year** | **Country** | **Study design** | **Sample size** | **Study population** | **Dietary intervention** | **Assessed outcomes of interest** | **Advantages** | **Disadvantages** |
| --- | --- | --- | --- | --- | --- | --- | --- | --- |
| Alasulami S et al.,2023 | Saudi Arabia | Prospective cohort study | 70 | 53-GDM women  7-PreDM | RF | -Risk of hypoglycemia  -Predictors of hypoglycemia  -Glycemic profile | -Fasting BSL reduced significantly | -Higher risk of hypoglycemia in T2DM  -T2DM was strong predictor of hypoglycemia  - No improvement in PP BSL |
| Abdulla S et al., 2023 | Karachi | Prospective case-control | 82 | 57-GDM, 25-healthy | RF | -Anthropometry  -Glycemic profile | -HbA1c significantly improved | - No changes in fasting BSL  - No significant reduction in BMI |
| Almogbel et al.,2022 | Australia | Retrospective cohort study | 345 | GDM women | RF | -Anthropometry  -Glycemic profile | -PP BSL improved | -No change in GWG  -Non-significant reduction in insulin treatment |
| Afandi B et al., 2017 | UAE | Prospective observational study | 32 | GDM women | RF | -Glycemic profile  -Risk of hypoglycemia  - Severity of hypoglycemia | - Mean glucose values lowered in RF but more reduction was seen in the RF+metformin group | -Risk and severity of hypoglycemia was seen in the RF+metformin group |
| Hassanein et al., 2021 | UAE | Prospective observational study | 25 | GDM women | RF | -Glycemic profile  -Risk of hypoglycemia | - Significant reduction in average mean glucose and HbA1c | -Hypoglycemia incidences increased  -Bodyweight increased |
| Afandi B et al., 2017 | UAE | Observational restropective study | 401 | GDM women | RF | -Glycemic profile  -Need for insulin | -Reduced HbA1c  -Decreased need for insulin | -Risk of hypoglycemia |
| **Fetal outcomes** | | | | | | | | |
| Abdulla S et al., 2023 | Karachi | Prospective case-control | 82 | 57-GDM, 25-healthy | RF | -Anthropometry  -APGAR score  - Neonatal BSL  -Neonatal serum bilirubin | - | -Head circumference significantly increased  -No changes in length, weight, MUAC and APGAR score  - Neonatal BSL and bilirubin levels increased non-significantly |
| Almogbel et al.,2022 | Australia | Retrospective cohort study | 345 | GDM women | RF | -Anthropometry  -Neonatal hypoglycemia  -Neonatal hyperbilirubinemia | -Neonatal hypoglycemia decreased with increased days of fasting | - Neonatal hyperbilirubinemia increased as the number of fasting days increased  -Risk of neonatal hyperbilirubinemia increased with >13 hours of fasting  - Risk of neonatal hypoglycemia increased with >15 hours of fasting  -Maternal RF during 2^nd^ and 3^rd^ trimester significantly increased the risk of neonatal hypoglycemia and hyperbilirubinemia  -Birthweight increased significantly  -Non-significant changes in incidences of macrosomia, LGA, SGA |

**Calorie restriction**

| **Author & Year** | **Country** | **Study design** | **Sample size** | **Study population** | **Dietary intervention** | **Assessed outcomes of interest** | **Advantages** | **Disadvantages** |
| --- | --- | --- | --- | --- | --- | --- | --- | --- |
| Tsirou, Efrosini et al., 2021 | Greece | Non-randomized, parrel, open-labeled feasibility study | 43 | GDM women (24-28^th^ GW) | 4 interventions  LED (1800kcal), LED+ Exercise, VLED (1600kcal), VLED+ Exercise | 1.Maternal anthropometry (GWG, MUAC)  2. Insulin use  3. C-section delivery  4. Gestational age  4. Depression  5. Urine ketones | 1. Total GWG was observed in LED (11.4 ± 5.0) compared to VLED (6.8 ± 7.6). MUAC reduced nonsignificantly in VLED expect for LED  2. The majority had GA between >37, <42 weeks in both groups no differences (LED- 16 (100%), VLED-14 (93.3%), P=0.48)  3. Non-significant differences in depression scores between all the groups | 1. The VLED group required insulin at 22 ± 8 GW compared to LED 25 ± 7. Required insulin units at labor was higher in VLED 26.8 ± 13.6 (p=0.36) compared to LED 18.9 ± 13.7. (p=0.27). No difference between insulin users with half of them requiring insulin  2. Both groups nonsignificantly had C-section delivery however proportion was higher VLED group (66.7%) compared to LED (50%), p=0.35.  3. 3 women from VLED group experienced mild, moderate, severe ketones compared to 2 women from LED group  4. Small sample size  5. 24 hr dietary recall  6. Recall/reporting bias |
| Hodson K et al., 2017 | UK | Mixed methods, case-control study | 16 | GDM women (21-34GW) | Low energy diet (1200kcal) | 1.Maternal Anthropometry (weight change)  2. Liver triacyglycerol  3. Glycemic profile  4. Lipid profile | 1. mean weight loss 0.4 ± 0.4 kg per week in LED diet compared to weight gain 0.3 ± 0.3 kg per week in control group (p<0.001). The total weight loss in participants was 1.6 ± 1.7 kg compared with a total gain of 1.4 ± 1.2 kg in comparators  2. Median liver triacylglycerol levels lowered significantly post-intervention 3.4% [IQR 1.1–4.1%] pre-diet, 1.4% [IQR 0.7–2.9%] post-diet; *p* = 0.006)  3. No participant in LED group required insulin therapy except for 2 women requiring metformin therapy  4. Lower percentage of women from the LED group required instrumental, elective, emergency c-sectional delivery compared to control group | 1. No change in fasting plasma glucose, post-prandial blood glucose levels, fasting insulin and HOMA2 index  2. No change in plasma triacylglycerol and lipid profile  3. Small sample size |
| Gray KL et al., 2021 | Australia | Randomized non-inferiority trial | 121 (61-IER, 60-CER) but 62 completed trial | Women diagnosed with GDM during previous pregnancy | Intermittent Energy restriction (VLED-500kcal for 2 consecutive days) vs continuous energy restriction (1500kcal/day) | 1.Maternal anthropometry  2. Glycemic profile  3. Physical activity and sleep quality | 1. At 12 mo, participants had lost an average (mean ± SD) of 4.3 ± 5.5% of their starting weight (IER, 5.0 ± 5.4%; CER, 3.5 ± 5.6%; P = 0.3) and 37% (n = 23) of participants had lost ≥5 kg (IER n = 13; CER n = 10; P = 0.6)  2. At 12 mo, HbA1c levels (n = 62), fasting plasma glucose levels (n = 51), fasting serum [insulin levels](https://www.sciencedirect.com/topics/nursing-and-health-professions/insulin-level) (n = 51), and HOMA-IR scores (n = 51) showed significant changes over time (P < 0.001), with no significant between-group differences  3. Weight loss was positively correlated with a reduction in fasting insulin levels, accounting for 9% of the variance in the change of fasting insulin levels (adjusted r^2^ = 0.07; P = 0.03). Weight loss also accounted for 9% of the variance in the change in HOMA-IR scores (adjusted r^2^ = 0.08; P = 0.03).  4. PA minutes decreased nonsignificantly over 12 months by 11 −11 ± 191 min for the IER group (n = 31) and −2 ± 269 min for the CER group (n = 27; P = 0.9). Non-significant improvement in overall sleep quality in both groups. | 1. High attrition rate  2. small sample size  3. Long term adherence to both the diets is challenging |
| **Fetal outcomes** | | | | | | | | |
| Tsirou, Efrosini et al., 2021 | Greece | Non-randomized, parrel, open-labeled feasibility study | 43 | GDM women (24-28^th^ GW) | 4 interventions  LED (1800kcal), LED+ Exercise, VLED (1600kcal), VLED+ Exercise | 1.Infant birth weight  2. Premature and stillbirth  3. SGA, LGA, AGA  4. Apgar score at 1 and 5 min | 1.Non significant changes in birthweight with lower BW in VLED group  2. 1 premature birth in VLED group (p=0.48)  3. No significant differences in SGA, LGA, majority were AGA between the groups (0.73)  4. Apgar score at 1 (p=0.55) and 5 min (p=1.00) did not differed significantly |  |
| Hodson K et al., 2017 | UK | Mixed methods, case-control study | 16 | GDM women (21-34GW) | Low energy diet (1200kcal) | 1. Birthweight  2. Special care admissions | 1. No significant differences in birthweight (LED-3360 ± 277, Control- 3361 ± 398, p=0.99)  2. No significant differences in special care admission (LED-1, Control 2, p=0.84) |  |

**Low-carb diet**

| **Author & Year** | **Country** | **Study design** | **Sample size** | **Study population** | **Dietary intervention** | **Assessed outcomes of interest** | **Advantages** | **Disadvantages** |  |
| --- | --- | --- | --- | --- | --- | --- | --- | --- | --- |
| Cui M et al., 2022 | China | Case-control | 265 (control= 113, intervention= 152) | GDM women (24^th^ -28^th^ GW) | Low-carb diet (40-50% Energy from carbs) vs control (60-65% E) | 1. Insulin treatment rate  2. Maternal lipid profile  3. Nutritional status  4. Glycemic profile  5. Anthropometric measures | 1. Delayed insulin requirement  2. Improved fasting BSL and 1-hr OGTT  3. No GWG | 1. No change in lipid profile and nutritional status (TP, ALB, HB) |  |
| Mijatovic Y et al., 2020 | Australia | RCT | 46 | GDM women (26^th^ -28^th^ GW) | Low carb (135g/day) vs Control (180-200g/day) | 1.Serum ketone  2. Glycemic profile  3. Anthropometry  4. Mode of delivery | 1. No significant change in ketones, HbA1c, fasting and post-prandial BSL |  |  |
| Rasmussen L et al., 2020 | Denmark | RCT | 12 | GDM (recently diagnosed) women | High carb vs Low carb for | 1. Maternal glycemic profile using CGM device | 1. Improved Glycemic variability and control in the low-carb group |  |  |
| Trout K et al., 2016 | USA | RCT | 68 | GDM women (24^th^-28^th^ GW) | Low-carb diet (30-40% Energy from carbs) vs control (50-55% E) | 1. Anthropometric measures  2. Glycemic profile  3. Insulin use  4. Use of oral medications  5. C-section delivery | 1. Non-significant reductions in gestational weight gain  2. Reduced fasting, and postprandial blood glucose levels, 3. Decreased need for insulin, use of oral medications  4. Reduced incidence of cesarean section |  |  |
| Moreno-Castillo C et al., 2013 | Spain | RCT | 152 | GDM women (24^th^-28^th^ GW) | Low-carb diet (40% Energy from carbs) vs control (55% E) | 1. Glycemic profile  2. Insulin use  3. GWG  4. Ketonuria  5. Maternal hypertension  6. C-section delivery | 1. low weight gain 1.4±2.0 vs 2.3±2.0 (p=0.017)  2. Reduced incidences of maternal hypertension (5.3%) vs 13.3% | 1. No change in fasting BSL, 1 and 2-hr postprandial glucose  Insulin (p=1.00)  Gestational age (39)  2. Moderate to high ketonuria  3. C-section delivery (33.8%) vs control 26.7% |  |
| Bao W et al., 2016 | US | Prospective cohort study | 722 | Women with a history of GDM | No intervention, FFQ data was analysed | 1.Development of T2DM | 1. LCD, high vegetable protein decreased risk of T2DM (1.29 (1.00–1.67) (P = 0.14 for trend). | 1.Low carb, high animal protein diet significantly increased risk of T2DM (2.18 (1.68–2.83), p<0.001)  2.Highest adherence to LCD increased risk of T2DM (2.13 (1.65–2.76), p<0.001) |  |
| **Fetal outcomes** | | | | | | | | | |
| Mijatovic Y et al., 2020 | Australia | RCT | 46 | GDM women (26^th^ -28^th^ GW) | Low carb (135g/day) vs Control (180-200g/day) | 1. Fetal anthropometry  2. Macrosomia | 1. Birthweight, SGA, LGA, macrosomia changed non-significantly  2. Reduced fat mass and increased fat-free mass |  |  |
| Trout K et al., 2016 | USA | RCT | 68 | GDM women (24^th^-28^th^ GW) | Low-carb diet (30-40% Energy from carbs) vs control (50-55% E) | 1. Anthropometry  2. Incidence of shoulder dystocia  3. Hypoglycemic events  4. Hospitalization | 1. Reduced hypoglycemic events | 1. Increased birthweight, head circumference, and abdominal girth (p>0.05)  2. Incidence of shoulder dystocia  3. Increased hospitalization |  |
| Moreno-Castillo C et al., 2013 | Spain | RCT | 152 | GDM women (24^th^-28^th^ GW) | Low-carb diet (40% Energy from carbs) vs control (55% E) | 1. Fetal anthropometry  2. Hypoglycemia | 1. Incidence of LGA (p=0.49), macrosomia (p=0.21) reduced  2. Incidence of neonatal hypoglycemia reduced (p=1.00) | 1. Incidence of SGA increased (p=0.47) |  |

**High Protein diet**

| **Author & Year** | **Country** | **Study design** | **Sample size** | **Study population** | **Dietary intervention** | **Assessed outcomes of interest** | **Advantages** | **Disadvantages** |
| --- | --- | --- | --- | --- | --- | --- | --- | --- |
| Trout K et al., 2022 | USA | RCT, cross-over design | 12 | GDM women | HP (30%) vs LP (15%) | -Glycemic and insulinemic response to meals | -PP glucose AUC values were significantly lower after HP diet than LP (p=0.01) on day 2.  -Lower PP mean glucose levels in HP diet than LP on day 2 at 30, 60, and 180 mins (p<0.05).  -Lower Insulin AUC values on day 1 with HP (p=0.005) after 3 hrs. | -Short trial (3 days)  -Altered composition of macronutrients (Carbohydrates) in High (CHO-35%, P=30%) and low (CHO-50%, p=15%) protein diet affects the interpretation. |
| Sarathi V et al., 2016 | India | RCT | 62 | GDM women | Soy protein vs high fibre diet | -Glycemic profile  -Need for insulin therapy | - Soy protein diet reduced post-prandial blood glucose levels.  - The need for insulin therapy at end of the intervention and at the time of delivery was lower in the soy-protein group. | -A high fiber diet significantly increased post-prandial blood glucose levels |
| Jamilian, M et al., 2015 | Iran | RCT | 68 | GDM women | Soy protein (70% plant + 30% animal protein) vs Standard diet (70% animal + 30% plant protein) | -Glycemic profile  -Insulin resistance and serum insulin | - Soy-protein diet significantly improved fasting plasma glucose, serum insulin, and insulin resistance | A standard diet with 70% animal protein and 30% plant protein significantly increased fasting plasma glucose, serum insulin levels, and insulin resistance (p<0.05) |
| **Fetal outcomes** | | | | | | | | |
| Sarathi V et al., 2016 | India | RCT | 62 | Newborns of GDM mothers | Soy protein vs high fibre diet | -Birth weight, LGA, Shoulder dystocia, neonatal hypoglycemia and TSH | -Birth weight was lower but within the normal range in the Soy group | - No significant changes in LGA, shoulder dystocia, neonatal hypoglycemia & TSH levels |
| Jamilian, M et al., 2015 | Iran | RCT | 68 | Newborns of GDM mothers | Soy protein (70% plant + 30% animal protein) vs Standard diet (70% animal + 30% plant protein) | -Preterm delivery, macrosomia, birth weight, length, head circumference, APGAR score, neonatal hypoglycemia | -Soy-protein diet significantly reduced neonatal hyperbilirubinemia | -No significant changes in preterm delivery, macrosomia, birth weight, length, head circumference, APGAR score at 1/5 mins, and neonatal hypoglycemia. |
| Maslova, E et al., 2017 | Denmark | Longitudinal Cohort Study | 1234 (Cases=608; Controls=626) | Newborns of GDM mothers | None | -Fasting insulin, HOMA-IR  -Abdominal obesity | -Fasting insulin & HOMA-IR were not associated with higher protein intakes | -Non-significant incidences of abdominal obesity were seen  - Higher intakes of red, processed meat, white meat, and dairy were associated with GDM. |
